# Validating the response surface method in entrepreneurship management research

**DOI:** 10.1016/j.mex.2021.101534

**Published:** 2021-09-30

**Authors:** Hsing-Er Lin, Dan K. Hsu, Michelle C. Hong, Yongchuan Shi

**Affiliations:** a National Sun Yat-Sen University, 70, LienHai Rd, Kaohsiung, 804, Taiwan; b North Dakota State University; 811 2^nd^ Ave N, Fargo, ND, 58102 USA; c Wenzhou University, Wenzhou, China

**Keywords:** Polynomial regression, Response surface method, Congruence effect test

## Abstract

This study adapts an existing methodology in psychology to assess congruence relationships in entrepreneurship management. More specifically, it describes the application of a response surface method to examine the congruence effect of two predictor variables on an outcome variable. The study presents both visual and text presentations to serve as a guideline that can aid management researchers in adapting the method. The paper underscores three strengths of using the response surface method as a robust analytical approach to evaluating congruent and incongruent relationships.

• The response surface method can be used to examine congruency and incongruency between variables in the field of management in general and entrepreneurship management in particular.
• The results can be visualized as two- and three-dimensional graphs.
• Compared with a traditional approach, the response surface method offers a clearer visual representation of a focal relationship.

## Graphic abstract

**Figure:**
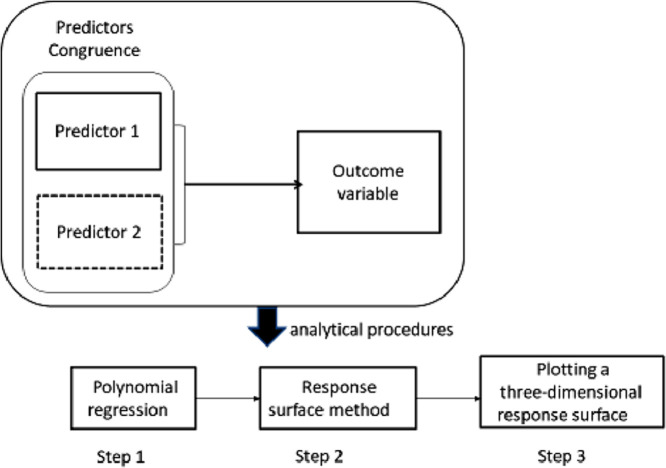

Specifications table

**Table:** 

Subject area:	Psychology
More specific subject area:	Ownership congruence effect tests in entrepreneurship management
Method name:	Response surface method
Name and reference of original method:	“When Ownership of the Venture Triggers Cofounders’ Unethical Pro-venture Behavior”, Journal of Business Venturing Insights, 2021, https://doi.org/10.1016/j.jbvi.2021.e00255
Resource availability:	Microsoft Excel.

## Introduction

Psychological factors play an important role in management research (e.g., [11]). In psychology, many variables are intertwined. Often, the congruence between two psychological predictors affects the outcome variable more than each predictor does alone [7]. The traditional approach to examine congruence between two variables in psychology was to construct an artificial variable by calculating the difference between the two variables. This approach was not applicable for two variables on different scales. The response surface methodology with advanced technique that allows accurate depiction and assessment of the three-dimensional surfaces corresponding to polynomial regression equations [4,^5^,^7^,8], offers more interpretive variance compared to the traditional approach. Moreover, the method “illustrates the relationships between focal variables more clearly” ([13], p.656). This technique is commonly used in studies examining congruence/incongruence of two variables in psychology (e.g., [8,14]) but still in its infancy in the field of entrepreneurship management. This paper provides a step-by-step tutorial for entrepreneurship management researchers to use the response surface method to examine congruent relationships between variables in the field of entrepreneurship management.

## Response surface method

Response surface methodology offers in-depth insight on the unique and collective effects of two predictors *A_1_* and *A_2_* [7,18] on outcome variable O through a visually represented three-dimensional model of the variables [18]. Congruence between *A_1_* and *A_2_* is denoted by the *A_1_* = *A_2_* diagonal line in the three-dimensional space, while incongruence is represented by the response surface pattern of O above the *A_1_* * *A_2_* floor [5].

This study applies polynomial regression. We compute and regress the outcome variable on the controls in Step 1. Subsequently, we regress on the controls, *A_1_, A_2_*, the square of *A_1_* (*A_1_^2^*), the cross-product of *A_1_* and *A_2_* (*A_1_* * *A_2_*), and the square of *A_2_* (*A_2_^2^*) in Step 2. Appropriateness of the response surface tests is denoted by a significant ΔR^2^ [4]. Following Edwards’ [4] approach, the estimation approach is as follows (control variables are included in regression equation but not displayed for simplicity):

$$O=b_{0}+b_{1}A_{1}+b_{2}A_{2}+b_{3}{A_{1}}^{2}+b_{4}\left(A_{1}A_{2}\right)+b_{5}{A_{2}}^{2}+e.$$

Next, we employ the estimated regression coefficients to plot the three-dimensional response surface, with *A_1_* and *A_2_* on the two horizontal axes and O on the vertical axis [14]. The horizontal plane comprises both the congruence (*A_1_* = *A_2_*) and incongruence (*A_1_* = –A_2_) diagonals. Congruence between the two predictors is denoted by the midpoint of the diagonal (i.e., *A_1_* = 0, *A_2_* = 0), while incongruent conditions are represented by the pattern along the diagonal from the midpoint to the two extremes. We adopt Edwards’ [4] approach to estimate the slopes of the congruent (*A_1_* = *A_2_* diagonal) and incongruent (*A_1_* = –*A_2_* diagonal) lines as *a_1_* = *b_1_* + *b_2_* and *a_3_* = *b_1_* – *b_2._* In addition, we determine the curvatures of the congruent and incongruent lines as *a_2_* = *b_3_* + *b_4_* + *b_5_* and *a_4_* = *b_3_* – *b_4_* + *b_5_*, where *a_3_* is the direction of incongruence (i.e., whether *A_1_* > A_2_ or *A_1_* < *A_2_*) [3,12] and *a_4_* denotes if the three-dimensional space takes the shape of a dome or bowl [2,6]. Thus, a hypothesis testing for the incongruence between A_1_ and A_2_ highlights only the coefficients for the incongruent line (*a_3_* and *a_4_*) as meaningful.

We empirically apply the method to examine the congruence–discrepancy effect between psychological ownership (PO) and equity ownership (EO) on unethical pro-venture behaviors (UPVB) [8]. More specifically, we analyze if the PO and EO of a cofounder triggers UPVB among other cofounders.

## Method validation

### Participants and steps

The application of the response surface method is demonstrated using field data collected to examine congruent relationships. Data are obtained for participants in an executive training program at a university in southeastern China. Given that Wenzhou is considered to be “a city of entrepreneurs” [16], we approached 220 venture founders who attended a training program and cofounded ventures in Wenzhou, China. We define cofounders as those who have set up a business venture in collaboration with one or more individuals [8]. According to Reynolds [17], more than 60% of new businesses are likely to shut down within five years [17]. Thus, the inclusion criteria are firms that were established within the past five years with one or more than one cofounder [8].

Following Podsakoff et al. [15], we collect data (survey 1 and 2) at different time points (time 1 and 2) to account for the problem of common method variance. Survey 1 administered at time 1 (September 2018) includes items related to PO and EO and individual demographic data [8]. Survey 2, conducted at time 2 (October 2018), addresses UPVB and collects individual demographic data. We offer a link to the online surveys for participants. 204 founders participated in survey 1, and 176 founders took survey 2. We eliminate founders who completed only one of the two surveys, leaving us with 151 founders and a 68.4% response rate. We further omit outliers (*N* = 12), defined as normal distributions outside three standard deviations of the mean for our key variables [8,19], achieving a final sample with founders from 139 firms. As indicated by other longitudinal studies, the level of attrition is acceptable [8,21].

A majority of the founders (77.3%) are male and report an average age of 26.6 years (*SD* = 7.5). Among them, 63.1% are single and 36.9% are married. The majority of the founders or 85.1% have a college degree, 12.1% have a master's or higher degree, and 2.8% have a high school degree. The studied founders set up (single handedly or collaboratively) an average of 1.6 ventures (including their current venture). Approximately three-quarter of the sample reports entrepreneurial experience between 1 and 44 years with an average of 3.4 years, and 37 founders (26.6%) have no prior entrepreneurial experience. In addition, 38 (27.3%) have at least one parent who was an entrepreneur. Approximately 136 (97.8%) of the sampled firms have more than one employee, with an average of 87 employees.

### Study variables

This study adopts scales that have been validated in the previous literature. We developed the survey items in English and then translated them into Chinese based on the suggestion of Brislin, Lonner, and Thorndike [1]. Any inconsistencies were discussed and resolved with minor modifications to fit the Chinese context.

We use UPVB as the outcome variable [8]. Given the unavailability of scales to examine UPVB, we “adapt scale items from existing research on unethical pro-organizational behavior” ([8], p.4; [20,22]). We select four items from Thau et al. [20] and one from Umphress, Bingham, and Mitchell [22] “relevant to the context of teams and modify the wording to fit the context of entrepreneurship” ([8], p.4). The revised items are as follows: “*Discredited another founder's performance to make the venture look better*,” “*To benefit the venture, I bad-mouthed another founder in the hope of getting him or her off the team*,” “*To benefit the venture, I provided misinformation to another founder whose behavior could have damaged the business*,” “*To benefit the venture, I concealed information from another founder whose behavior could have damaged the business*,” and “*I deliberately excluded another founder because I thought he or she would diminish the value of the team*.” ([8], p.4). Participants responded with the frequency of occurrence for each item on a seven-point Likert scale, where 1 denotes “none” and 7 is “very often.” Cronbach's alpha for this measure is .84.

The predictors for this study are PO and EO. We adapt Van Dyne and Pierce's [23] four-item scale to the present research context. A sample item is “*I feel a very high degree of personal ownership toward this venture.*” ([8], p.4). Participants rated each item on a seven-point Likert scale, where 1 denotes “strongly disagree” and 7 is “strongly agree.” Cronbach's alpha of this measure is .89. We measure “EO by asking cofounders to self-report the percentage of their equity ownership” (i.e., “*How much equity do you own in your current venture?*”) ([8], p.4).

Literature indicated that subsequent behaviors can be influenced by whether entrepreneurs have entrepreneurial experiences [9], and unethical behaviors are more likely to be observed with an increase in team size [8,10]. Thus, we accounted for the number of founders in the founding team and years of entrepreneurial experience to control for confounding effects on dependent variables [8]. We do not include age because it is highly correlated with years of experience. We exclude also founders who were sole owners of their venture since they are not the study's target group. We standardize the scores for the two variables prior to the regression analyses to facilitate a comparison between estimates.

### Analysis procedure of response surface method

As previously mentioned, the polynomial regression is applied to regress the outcome variable UPVB on the controls in Step 1 and the outcome variable on controls *A_1_* (psychological ownership denoted as PO), *A_2_* (equity ownership denoted as EO), the square of *A_1_* (*A_1_^2^*), the cross-product of *A_1_* and *A_2_* (*A_1_* * *A_2_*), and the square of *A_2_* (*A_2_^2^*) in Step 2. We adopt the following estimation (control variables are included in regression equation but are not displayed for simplicity) ([8], p.8):

$${}^{\prime \prime }\text{UPVB}=b_{0}+b_{1}\text{PO}+b_{2}\text{EO}+b_{3}PO^{2}+b_{4}\left(\text{PO}*\text{EO}\right)+b_{5}EO^{2}+e.^{\prime \prime }$$

**Fig. 1:**
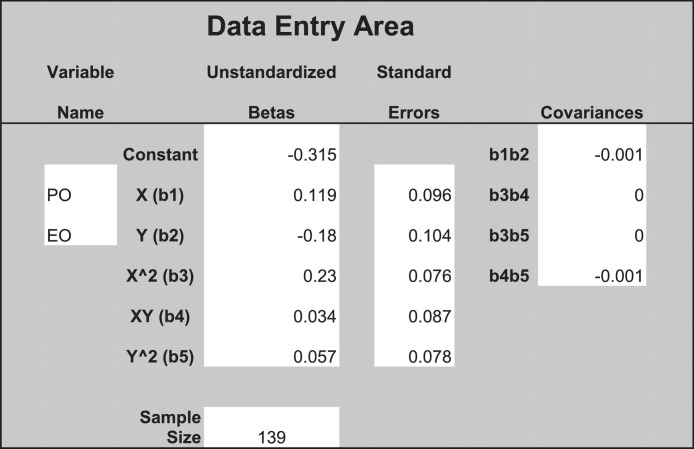
The results of the unstandardized regression coefficients, associated standard errors, and the covariances in the Excel sheet.

**Fig. 2:**
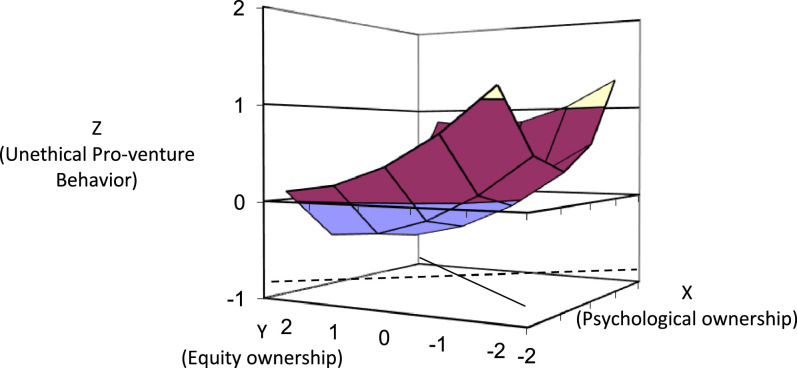
Response Surface for Congruence between PO and EO.

**Fig. 3:**
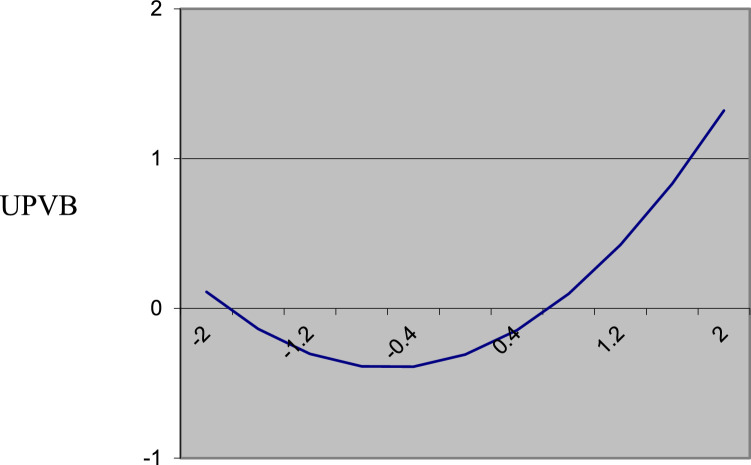
Two-dimensional surface between PO and EO along the incongruence line.

Table 1 shows the results of the polynomial regression for UPVB. We include the polynomial regression higher-order terms. The results for ΔR^2^ (0.088, *p* = 0.043 < 0.05) indicate that the response surface method is an appropriate approach to examine the ownership discrepancy hypothesis [8]. We conduct the response surface analysis using the syntax in Microsoft Excel developed by Shanock et al. [18]. We entered the results of the unstandardized regression coefficients, associated standard errors, and the covariances from the polynomial regression under “Data Entry Area” in the Excel sheet as shown in Fig. 1. Fig. 2 presents the response surface for UPVB on the basis of the estimated coefficients from the polynomial regression model and response surface test. Our hypothesis is that UPVB increases to the extent that cofounders’ PO higher than their EO. We test this hypothesis according to the slope (*a_3_*) along the incongruence diagonal (PO = −EO). Table 1 shows that the slope of the incongruence diagonal (PO = −EO) is positive and significant (*a_3_* = 0.30, *p* ≤ 0.05). In other words, UPVB increases with the widening of the gap between PO and EO, where the former higher than the latter. Further, the curvature along the incongruence line (*a_4_*) is positive and significant at the moderate level (*a_4_* = 0.26, *p* ≤ 0.10), indicating that the surface is bowl shape but not perfect. In Fig. 3, we plot the two-dimensional surface between PO and EO along the incongruence line to further explore the surface shape. UPVB is the lowest when EO is equivalent to PO and increases when PO or EO higher than each other. The right-hand side of the midpoint indicates that UPVB increases as a nonlinear function of PO > EO and confirms the findings from *a_3_*. These results support the hypothesis.

**Table 1:** Results of Polynomial Regression.

	DV: Unethical Pro-venture Behavior		
	*b*	SE	*p*-value
Constant	-0.308	0.134	0.023
Control variables			
Number of founders	0.185	0.087	0.035
Entrepreneurial experience	-0.080	0.086	0.350
Polynomial terms			
b_1_: Psychological ownership (PO)	0.118	0.095	0.213
b_2_: Equity ownership (EO)	–0.185	0.103	0.074
b_3_: PO ^2^	0.230	0.075	0.002
b_4_: PO × EO	0.034	0.085	0.691
b_5_: EO ^2^	0.060	0.077	0.438
R^2^	0.106		
∆R^2^	0.088		0.043
Response surface tests			
Congruence line (A_P_ = A_E_)			
*a_1_*: Slope (b_1_ + b_2_)	–0.07	0.13	0.615
*a_2_*: Curvature (b_3_ + b_4_ + b_5_)	0.32	0.13	0.014*
Incongruence line (A_P_ = –A_E_)			
*a_3_*: Slope (b_1_ – b_2_)	0.30	0.15	0.041*
*a_4_*: Curvature (b_3_ – b_4_ + b_5_)	0.26	0.14	0.078^†^
F for the three quadratic terms	2.154	0.946	

Note: b= unstandardized regression coefficient. *n* = 139. *p < .05. †*p* < .10

### Robustness check

Additional evidence for the hypothesis can be derived from examining the surface shape along the congruence line. Our results show that UPVB is least likely to occur when PO is congruent with EO. As shown in Fig. 2, the lowest point on the response surface along the congruence line is around the midpoint. Table 1 shows that the curvature of the congruence diagonal is positive and significant (*a_2_* = 0.32, *p* = 0.014<0.05), suggesting a bowl-shaped curvilinear relationship between EO-PO and UPVB. Specifically, UPVB is higher when EO-PO is congruent at high [PO = 2, EO = 2; UPVB = 0.85] or low levels [PO = -2, EO = -2; UPVB = 1.12] compared to a moderate level, i.e., incongruence [PO = 0, EO = 0, UPVB = -0.31]. These analyses and Fig. 2 provide additional evidence for a curvilinear relationship among EO, PO, and UPVB.

## Conclusion

The use of the response surface method to examine congruent relationships between variables is still in its infancy in the field of entrepreneurship management. This paper demonstrates this method empirically by applying the method to test whether cofounders’ UPVBs were incited by the congruence/discrepancy effect of PO and EO. Using this method, we are able to understand profoundly for the unique and collective effects between PO and EO under the conditions of (in) congruence. By doing so, our paper makes a methodological contribution to the literature on the relationship between the ventures’ ownership and their cofounders’ UPVB. Future research may replicate this method to test two different predictors, such as social identity and personal identity, on UPVB or other outcome variables.
